# Supplementary material for: Incidence of lung cancer and mortality among civil construction industry workers: A protocol for a systematic review and meta-analysis
Source: PLoS One. 2021 Apr 26;16(4):e0250377. doi: 10.1371/journal.pone.0250377 (PMC8075260; doi:10.1371/journal.pone.0250377)
Supplement: Attachment — Submitted filename: Response to Reviewers.docx [file pone.0250377.s002.docx]

30.01.2021

Prof. Rakibul M Islam

Academic Editor

**PLOS ONE**

Re: **PLOS ONE Decision: Revision required [PONE-D-20-29581]**

Dear Prof. Rakibul,

We shall be very much appreciated if you may consider the revised version of our paper entitled (former title) " **Lung cancer risk in workers of the civil construction industry: A protocol for a systematic review and meta-analysis**” by *Schnorr and colleagues,* suitable for publication in PLOS ONE. Please find below point-by-point responses to the referee’s comments.

We greatly appreciated the editor´s and reviewer’s comments and suggestions. In this version, we performed the revision of our manuscript according to all their requests (please, kindly find the letter below). We do hope that all the present corrections could attend the editor and reviewer’s requirements for publication in PLOS ONE.

With best regards.

Yours sincerely,

Carlos Eduardo Schnorr (on behalf of all authors)

**EDITORS' COMMENTS**

*Thank you for submitting your manuscript to PLOS ONE. After careful consideration, we feel that it has merit but does not fully meet PLOS ONE’s publication criteria as it currently stands. Therefore, we invite you to submit a revised version of the manuscript that addresses the points raised during the review process.*

*1. The reviewers rightly pointed out the vagueness of the protocol objective. The review authors stated “the main objective of our systematic review and meta-analysis is to determine how does working in the civil construction industry impacts on lung cancer risk for workers” which is not clear what they intend to do. Authors’ three research questions telling exactly the same thing in different ways. They are in fact examining an association between occupational exposure in the civil construction industry and lung cancer. They need to clarify whether they are assessing lung cancer events or lung cancer mortality or both.*

**The authors:** We are sorry about the vagueness of the protocol objective. We have now revised the main objective and the research questions in order to clarify that we will be assessing lung cancer risk through both mortality and incidence outcomes.

*2. Electronic searches. It would be relevant to consider Medline instead of PubMed.*

**The authors:** We have considered Pubmed instead of Medline because Pubmed already include the MEDLINE database plus the many other types of citations, as described at the US National Medicine Library website (https://www.nlm.nih.gov/bsd/difference.html).

*3. Authors are using funnel plot to report publication bias. However, the funnel plot only provides visual impressions. So the author should use Egger's test as well for testing publication bias.*

**The authors:** We had predicted the use of funnel plots to assess publication bias as “Publication bias will be investigated by visual inspection of Begg’s funnel plots which is a scatter plot of the studies that is constructed from a meta-analysis”. However, we have now also added Egger’s regression test to our analysis plan in “We will also perform Egger’s regression tests if ten or more studies are included in a meta-analysis”.

*4. Give the version of RevMan that will be used: the latest "5.3"?*

**The authors:** We update the information in the manuscript, and we will use RevMan 5.4.1, the latest version released in September 2020, as stated at Cochrane Training website (https://training.cochrane.org/online-learning/core-software-cochrane-reviews/revman).

*5. Discuss the dissemination of findings in the Discussion section.*

**The authors:** We have included the dissemination of findings in the Discussion section. We plan to disseminate the results of this systematic review and metanalysis to researchers and research funders in scientific conferences and by publication in a peer-review journal relevant to the field, as well as to policymakers by publication of print materials and personal communication.

**REVIEWERS' COMMENTS**

**REVIEWER #1:**

*Manuscript title: Lung cancer risk in workers of the civil construction industry: A protocol for a systematic review and meta-analysis*

***A) Title***

*The Authors tried to address the Lung cancer risk in workers of the civil construction industry. Later, they will pool the incidence of type of lung cancer among these populations and the title should be modified in this context.*

*Suggestion: “**Incidence of lung cancer and mortality among civil construction industry workers: A protocol for a systematic review and meta-analysis”*

**The authors:** We very much appreciated the reviewer´s suggestion, and the title has been modified to better describe the context of the proposed protocol for systematic review and meta-analysis. The title changed from “Lung cancer risk in workers of the civil construction industry: A protocol for a systematic review and meta-analysis” to “Incidence of lung cancer and mortality among civil construction industry workers: A protocol for a systematic review and meta-analysis.”

***B) Background***

*The authors didn’t show the problem associated with exposure to carcinogenic substances. What are these chemicals? What have been done so far? What are the gaps identified? What this systematic review going to investigate? How this review is different from the previous studies?*

*The findings of previous observational studies were consistent, but the authors considered the justification as there is no systematic review on this title. Do you think absence of systematic review must be the justification to carry out systematic review? To my understanding, systematic review should be conducted if:*

*- The existing studies are contradicting*

*- Existing studies recommending*

*- New studies are coming out*

*I have tried to see some literatures on incidence of /exposure risk as you mentioned/ lung cancer associated with chemical exposure among industrial workers, but there are a couple of reviews and systematic reviews on specific chemical exposure and risk of cancer among these populations. For example, Alonso-Sandra et al: Association between Occupational Exposure to Wood Dust and Cancer: A Systematic Review and Meta-Analysis; Danato et al: Mortality and cancer morbidity among cement production workers: a meta-analysis; Boschman et al: Occupational demands and health effects for bricklayers and construction supervisors: A systematic review and some others reporting the incidence of lung cancer as well.*

**The authors:** Thank you for all your observations and questions. They have helped us to improve the description and justification of the proposed systematic review and meta-analysis.

First, we had expanded the information about the problem associated with exposure to carcinogenic substances in the construction industry. We have included information about the physical and chemical agents commonly found in the construction industry environment and a short review of the state of art and knowledge gap about the subject. We also included information about previous primary studies and systematic reviews, as well as arguments that justify our proposal for a systematic review and meta-analysis. We have tried to clarify in the manuscript why we believe the reviews and systematic reviews on specific chemical exposure and risk of cancer already published failed to answer our research question.

Second, we respectfully disagree with the reviewer´s opinion about the consistency of previous observational studies. It seems that, in the absence of a systematic review, the conclusion of consistent evidence is based only in the number of positive or negative results in the literature, but this cannot objectively determine the strength of the evidence. We consider the systematic review and metanalysis as a far superior method in comparison with this vote-counting method used to synthesize available studies. Additionally, a systematic review and metanalysis will help us to develop a more correct estimate of effect magnitude and to provide a more complex analysis of harm associated with the work in the construction industry. Thus, the absence of systematic reviews is not the only justification for our systematic review and metanalysis.

Third, we would like to clarify that all studies and reviews cited address different research questions. The first review addresses the effects of the exposure to wood dust in different settings and populations to also include a single occupation in the construction industry. The second review addresses the same outcome (cancer and mortality), but in a completely different population and exposure setting (the cement industry workers vs construction industry workers). The third review addresses a single occupation in the construction industry and does not allow for comparison between different occupations in the construction industry. The construction industry is a very complex industry with many different occupations, including but not limited to carpenters, floor layers, bricklayers, painters, electricians, plumbers, welders, scaffolders, roofers, masons, sheet metal workers, rebar workers, construction laborers, machine operators, drywall installers, and insulators. In our opinion a systematic review covering the most occupations in this industry will be a valuable tool to identify risk differences in the setting.

***C) Method and material***

*- I would like to appreciate the authors for their tremendous work on method. It was coherent, detailed, and interesting methodology but I have some minor comments: PICOs seems randomized trials. You are going to include studies not participants and you don’t need to mention gender, but studies conducted on certain age, gender, etc.*

*- Why don’t you include prospective Cohort, comparative cross-sectional and randomized trials*

**The authors:** We are glad to know that we have described the methods somewhat in details. Thank you for contributing with crucial points. They were all fundamental for the substantial improvement and transparency of our methodology.

First, we have reviewed the text to make sure that the acronym PICOs is not mentioned, since we do not wish to include interventions, but naturally occurring exposures. We have used, however, a similar structure to describe our methods. Observational studies of exposure are similar to trials when describing population, exposure (not intervention), control, outcome, and study design. The main difference is that because of the harmful nature of the exposure hereby studied, it would be unethical to conduct or include randomized clinical trials.

Second, we have corrected now the writing describing the studies to be included (not participants in the studies). Corrected as follows: “We will include studies that investigate effects on participants (both male and female) who are construction workers, with unequivocal evidence of occupational exposure to chemical pollutants, such as, but not limited to: carpenters, floor layers, bricklayers, painters, electricians, plumbers, welders, scaffolders, roofers, masons, sheet metal workers, rebar workers, construction laborers, machine operators, drywall installers, and insulators. No lower or upper limits to the age of the participants included in the studies will be set.”

Finally, we have included all kind of cohort studies and comparative cross-sectional. Although it is not our intention to include clinical trials due to the possibly toxic and harmful nature of the exposure we are investigating.

*- Your pan of data analysis will be with review manager, but you plan to perform regression analysis, how come? Review manager can’t perform regression, but R and STATA and other software can.*

**The authors:** This is surely the case. We will conduct regression analysis using the package *meta* in the R environment. We have now added this to the manuscript.

*- Is publication bias not your issue??*

**The authors:** We had predicted the use of funnel plots to assess publication bias as “Publication bias will be investigated by visual inspection of Begg’s funnel plots which is a scatter plot of the studies that is constructed from a meta-analysis”. However, we have now also added Egger’s regression test to our analysis plan in “We will also perform Egger’s regression tests if ten or more studies are included in a meta-analysis”.

*- What about your pan on overall quality of evidence? You don’t use the GRADEpro?*

**The authors:** We had described the quality analysis as “Two reviewers will independently screen articles, extract data, and assess scientific quality using standardized forms and published quality assessment tools tailored for each study design.” However, we agree that this description was vague. And this is because we cannot use the ROBINS-I or RoB 2.0 tools because they are design for interventional studies. The ROBINS-E tool is unfortunately not available at the moment, but it is currently being developed by researchers at University of Bristol (UK), McMaster University (Canada) and the Environmental Protection Agency (USA) (https://www.bristol.ac.uk/population-health-sciences/centres/cresyda/barr/riskofbias/robins-e/). The New-Castle Ottawa tool is one of the most used in the past, but it is clear that the tool is quite subjective. In this sense, we have now included the following: “(ROBINS-E tool will be used in case it is already available at https://www.bristol.ac.uk/population-health-sciences/centres/cresyda/barr/riskofbias/robins-e/. Otherwise, the New-Castle Ottawa rating scale will be used. Any of those will be also used in combination with the GRADE approach for quality of evidence through GRADEpro – https://gdt.gradepro.org/app/).”

***D) Discussion***

*- The plan of discussion is very narrow*

*- How will you compare your systematic review with the existing reviews?*

*- What do you think will be the political and health care implication of your study?*

**The authors:** We have improved the discussion section of the manuscript. We have added information about lung cancer epidemiology and about future comparison with previous systematic reviews. We also added information about the future implication of our study.

**REVIEWER #2:**

*Dear Editor,*

*Thank you for providing me the opportunity to review this protocol. The protocol is well devised and covers most aspects needed to answer the study question. However, I have some comments listed below:*

***A) Introduction***

*• Line 45 and 49: The authors should include more recent references for the statistics if available.*

**The authors:** We have updated the statistics as requested by the reviewer. We updated the construction industry share of the global workforce to 7% (Sertyeşilışık, 2017) and the data on construction industry energy consumption to 39% (United Nations & International Energy Agency, 2020). We also now present data about the carbon dioxide emissions related to construction sector activities (United Nations & International Energy Agency, 2020).

*• Line 67: Could the authors be clear in presenting if the finding is of a primary study or a review paper?*

**The authors:** We have revised all studies citation to ensure to be clear about the primary studies and review papers cited along the manuscript. However, the specific finding in Line 67 was eliminated from manuscript through the major revisions requested by reviewer #1.

***B) Review Objectives***

*• Line 88: The protocol does not appear to state the main objective precisely. Is the object to determine "how does it impact" or "quantification of the risk if analyzed". The language of the objective might create confusion.*

**The authors:** Our main objective is to summarize the evidence on the impacts of construction work on lung cancer risk. We have now stated it more clearly, specifying that a meta-analysis will be conducted provided that enough studies are included in the systematic review (lines 115 to 173).

***C) Methods/design***

*• Line 101: The authors should revise the PROSPERO protocol to match with the submitted manuscript. Please provide the dates of the planned search in the abstract at the end of line 23 as from date 'MM/YYYY' to date 'MM/YYYY' (is it until November 19, 2019?) If so, the authors should be able to provide an adapted PubMed search strategy with the number of studies retrieved alongside as an appendix.*

**The authors:** Thank you for all considerations on method. They have helped to improve the description and transparency of the manuscript greatly. Indeed, we have failed to the updates published in our PROSPERO protocol in time. It should soon be online and in full agreement with the present manuscript. We are awaiting PROSPERO’s evaluation.

Searches will be conducted from inception and updated to at most six months before the submission of the final manuscript. This information has been added in the end of the “Searches section” of the methods at lines 200-202 and also included in the abstract.

***D) Participants/Population***

*• Line 123: Age restriction is excluded in the manuscript which is inconsistent with the PROSPERO protocol. Please ensure uniformity.*

**The authors:** We have ensured that the PROSPERO protocol is updated accordingly.

***E) Search Strategy***

*• Line 150: I would recommend that authors also search Embase database (as mentioned in PROSPERO protocol). Else, the search might result limited studies.*

**The authors:** We have now also included Embase as one of the databases in which we will conduct the search.

***F) Study selection***

*• Line 170: Mention the reference manager "XXX" at the first use rather than below.*

**The authors:** Thank you for this comment. We have decided to use the Rayyan platform, now cited in the text.

*• Line 179: Use name "PRISMA flow diagram" rather than flow chart.*

**The authors:** This has been corrected accordingly.

***G) Other comments***

*• How will the authors evaluate the quality of evidence? Do they plan to use any tool?*

**The authors:** We had described the quality analysis as “Two reviewers will independently screen articles, extract data, and assess scientific quality using standardized forms and published quality assessment tools tailored for each study design.” However, we agree that this description was vague. And this is because we cannot use the ROBINS-I or RoB 2.0 tools because they are design for interventional studies. The ROBINS-E tool is unfortunately not available at the moment but is currently being developed by researchers at University of Bristol (UK), McMaster University (Canada) and the Environmental Protection Agency (USA) (https://www.bristol.ac.uk/population-health-sciences/centres/cresyda/barr/riskofbias/robins-e/). The New-Castle Ottawa tool is one of the most used in the past, but it is clear that the tool is quite subjective. In this sense, we have now included the following: “(ROBINS-E tool will be used in case it is already available at https://www.bristol.ac.uk/population-health-sciences/centres/cresyda/barr/riskofbias/robins-e/. Otherwise the New-Castle Ottawa rating scale will be used. Any of those will be also used in combination with the GRADE approach for quality of evidence through GRADEpro – https://gdt.gradepro.org/app/).”

*• What are the criteria under which data will be synthesized? (e.g; minimum number of studies to pool the results) In line 212, authors state "extremely high heterogeneity" which is unclear. What is the level of consistency required for synthesis in this review (< 75% or <85% or something else?)*

**The authors:** We have now included a minimum of three studies to pool the results in a meta-analysis (line 246). A minimum of ten studies was added for us to be able to perform subgroup and meta-regression analyses (lines 261-262) as well as for performing the Egger’s regression test for publication bias (line 256).

*• The authors should include a sub heading Dissemination or combine Ethics and Dissemination.*

**The authors:** We have added a subheading stating the following: “The present study does not require ethical approval, since the original studies must have been conducting according to each local ethics committee. We plan to disseminate the results of this study in scientific conferences and by publication in a peer-review journal relevant to the field.”

***H) Optional suggestions to authors***

*The manuscript appears to be comprehensive, but the authors need to improve some common errors in language which hampers the readability of the paper (e.g; use of full forms more than once, minor spelling errors).*

**The authors:** We have revised the whole manuscript in order to improve language and find potential typographical mistakes. We sincerely apologize for all language errors. With all, in case our new text might not satisfy the referee, we are willing to submit the whole manuscript to be reviewed by a premium quality international English-language editing service.
