# Supplementary material for: Incidence of lung cancer and mortality among civil construction industry workers: A protocol for a systematic review and meta-analysis
Source: PLoS One. 2021 Apr 26;16(4):e0250377. doi: 10.1371/journal.pone.0250377 (PMC8075260; doi:10.1371/journal.pone.0250377)
Supplement: Attachment — Submitted filename: Response to Reviewers.docx [file pone.0250377.s003.docx]

15.03.2021

Prof. Rakibul M Islam

Academic Editor

**PLOS ONE**

Re: **PLOS ONE Decision: Revision required [PONE-D-20-29581]**

Dear Prof. Rakibul,

We shall be very much appreciated if you may consider the revised version of our paper entitled (former title) " **Lung cancer risk in workers of the civil construction industry: A protocol for a systematic review and meta-analysis**” by *Schnorr and colleagues,* suitable for publication in PLOS ONE. Please find below point-by-point responses to the last editor’s and referee’s comments. We do hope we now could attend to all editor and reviewer’s requirements for publication in PLOS ONE.

With best regards.

Yours sincerely,

Carlos Eduardo Schnorr (on behalf of all authors)

**EDITORS' COMMENTS**

*Please review your reference list to ensure that it is complete and correct. If you have cited papers that have been retracted, please include the rationale for doing so in the manuscript text, or remove these references and replace them with relevant current references. Any changes to the reference list should be mentioned in the rebuttal letter that accompanies your revised manuscript. If you need to cite a retracted article, indicate the article’s retracted status in the References list and also include a citation and full reference for the retraction notice.*

**The authors:** We have reviewed the reference list text to make sure that it is complete and correct. We doble checked each reference from the list in PubMed and Retraction Watch Database (<http://retractiondatabase.org/>) and found no retracted papers in our reference list.

However, we found and included one erratum for the reference “Consonni D, De Matteis S, Pesatori AC, Cattaneo A, Cavallo DM, Lubin JH, et al. Increased lung cancer risk among bricklayers in an Italian population-based case-control study. Am J Ind Med. 2012 May;55(5):423–8.” Both the original and the erratum are now cited in the manuscript. We also revised the reference for the Cochrane Handbook for Systematic Reviews of Interventions. We now correctly cite the handbook as “Higgins JP, Green S, editors. Cochrane Handbook for Systematic Reviews of Interventions Version 5.1.0 [updated March 2011]. 2011. 639 p.” Finally, we found one missing reference in the manuscript (line 288) and the reference is now cited as “British Health and Safety Executive. Occupational Cancer statistics in Great Britain, 2020. London; 2020.”

**REVIEWERS' COMMENTS**

**REVIEWER #1:**

*I would appreciate the authors for their tremendous work and rigorous response on all concerns being forwarded. I have tried to see the manuscript thoroughly and it was well improved substantially. Finally, I would recommend publication in PLOS ONE.*

**The authors:** Thank you for all your previous observations and questions. They have helped us to improve the manuscript of the proposed systematic review and meta-analysis.

**REVIEWER #2:**

*The manuscript is well revised to improve the transparency, clarity and reproducibility of the planned work. The authors have addressed the comments and the responses are clear. However, I still have some minor comments listed below:*

***A) Ethics***

*• I would recommend modifying the tone of Ethics statement to make it sound better. It can be toned as "This review does not require ethical approval as the review is entirely based on the published data of the ethically approved primary studies."*

**The authors:** We very much appreciated the reviewer´s suggestion, and the Ethics statement has been modified to sound better. The Ethics statement changed from “The present study does not require ethical approval since the original studies must have been conducting according to each local ethics committee.” to “This review does not require ethical approval as the review is entirely based on the published data of the ethically approved primary studies.”

***B) Study selection***

*• Line 190: "where agreement cannot be reached” is dispensable.*

**The authors:** This has been corrected accordingly.

***C) Discussion***

*• Improvement on discussion section is fitting. However, it feels as the authors have too many introductory statements on lung cancer evidence which is not relevant here (Line 287-295). How does the evidence provided here link up with the advantages of the paper presented thereafter as "In this way, the results obtained from our study will..."? I would recommend summarising it. Introduction section should be better for presenting the facts and statistics on the grave nature of the outcome due to the risks among construction industry workers.*

**The authors:** Thank you for all your observations and suggestions. We concentrated the facts and statistics in the introduction section. And we have summarized the statements on lung cancer evidence in the Discussion section, to improve the section clarity and content.

*• The dissemination part is well addressed and can be a separate paragraph.*

**The authors:** This has been revised accordingly.
